# Opportunities to Optimize Outcomes of Diagnosis and Treatment of HIV and Syphilis in Pregnancy: the Quest to Eliminate Maternal and Vertical Transmission

**DOI:** 10.1007/s11904-025-00739-y

**Published:** 2025-04-23

**Authors:** Dvora Joseph Davey, Alex de Voux, Natalie Shaetonhodi, Michael Marks, Lisa Frigati, Tendesayi Kufa

**Affiliations:** 1 University of California Los Angeles, Los Angeles, USA; 2 Division of Epidemiology & Biostatistics, School of Public Health, University of Cape Town, Cape Town, South Africa; 3 Clinical Research Department, Faculty of Infectious and Tropical Diseases, London School of Hygiene and Tropical Medicine, London, United Kingdom; 4 Department of Pediatrics and Child Health, Stellenbosch University, Cape Town, South Africa; 5 National Institute of Communicable Diseases, Centre for HIV/STIs, Johannesburg, South Africa; 6 School of Public Health, University of the Witwatersrand, Johannesburg, 2131 South Africa

## Abstract

**Background:**

There is an urgent need to improve interventions for HIV and syphilis in pregnancy to achieve elimination.

**Results:**

The tenets of vertical transmission strategies for HIV and syphilis overlap but have varying success due to differences in their transmission dynamics, diagnoses, and treatment. Key principles include prevention of maternal infection, screening and diagnosis early and throughout antenatal care, curative treatment (syphilis), viral load suppression (HIV), early infant diagnosis and treatment (HIV and congenital syphilis). We recommend improved guidelines, provider training and focused research and surveillance, including implementation studies to align HIV and syphilis screening and treatment during pregnancy. Opportunities to integrate syphilis screening and treatment into antenatal and HIV care enable providers to offer comprehensive maternal care.

**Conclusion:**

Integrated HIV/syphilis services ensure a cohesive and person-centered approach, improving health outcomes through streamlined, efficient, and family-centered care pathways. We recommend key interventions to reduce HIV and syphilis in pregnancy and prevent vertical transmission.

**Supplementary Information:**

The online version contains supplementary material available at 10.1007/s11904-025-00739-y.

## Introduction

HIV and syphilis infections during pregnancy pose critical public health challenges, significantly affecting both maternal and child health. The World Health Organization (WHO) estimates that in 2023, 1.2 million pregnant women were living with HIV (WLHIV) [1] and 120,000 children acquired HIV, the majority through vertical transmission (VT) [2]. Globally, the estimated prevalence of maternal syphilis in 2016 was 0.69 percent (95% CI = 0.57–0.81) [3]. In 2022 there were 523 cases of congenital syphilis (CS) per 100,000 live births [4], over ten times the WHO target of 50 cases per 100,000 live births for elimination of CS.

### HIV in Pregnancy

Over half of HIV infections occur among cisgender women with notably high incidence among pregnant and lactating women (PLW) [5, 6]. Increased risk of HIV acquisition during gestation due to biological and behavioural changes coupled with potential for VT make HIV prevention among PLW a unique and critical global health priority [6, 7].

Women face an unacceptably elevated risk of HIV acquisition during pregnancy and breastfeeding [6]. HIV incidence during pregnancy and breastfeeding in Africa was estimated at 3.6/100 person-years between 2006–2017 and 4.2/100 person-years in southern Africa [5] and acute maternal HIV infection is associated with increased risk of VT [8]. Incident HIV in pregnancy and breastfeeding accounts for one-third of VT.

PLW require effective, female-controlled interventions to prevent HIV acquisition during pregnancy and breastfeeding [9]. Pre-exposure prophylaxis (PrEP) is a safe and effective tool for prevention of HIV in women at elevated risk [10]. The most widely available formulation of PrEP, an oral pill taken daily, presents challenges in persistence and adherence among PLW that undermine its prevention effectiveness [11, 12]. More promising long-acting injectable PrEP formulations (Cabotegravir and Lenacapavir) promise to overcome these challenges and must be made available to all PLW at risk of HIV acquisition prior to, during and after pregnancy (especially whilst breastfeeding) [13].

Untreated HIV can result in maternal morbidity, mortality, miscarriage, and preterm birth [14]. Between 2015 to 2023, global ART coverage among PLW plateaued around 84% [15], far below the second UNAIDS 95-95-95 target for elimination of VT. Global estimates of viral suppression (VS) among pregnant women at labor and delivery are lacking [16], with limited data from Africa reporting variable VS rates (<200 copies/ml) among pregnant women (56%–96%) [16–19]. Younger women, those not on ART at their first antenatal visit, and those with poor adherence are more likely to be viremic [16–20]. Expanding HIV treatment and adherence support to achieve VS, combined with primary prevention measures including oral and injectable PrEP, could accelerate progress toward the UNAIDS goal of reducing new HIV infections to below 375,000 and eliminating VT by 2025 [21].

### Syphilis in Pregnancy

Syphilis is caused by the bacterium *Treponema pallidum*, subspecies, *pallidum* which when left untreated will progress through a series of clinical stages. Syphilis is most infectious during the primary and secondary stages (early syphilis) characterized by clinical symptoms such as a painless, usually solitary, ulcerative lesion and lesions on the palms of the hands or the soles of the feet [22]. Globally approximately half of syphilis cases occur in cisgender women aged 15–49 [23]. VT of syphilis occurs via the transplacental route during pregnancy [24]. Although syphilis is treatable, the disease has seen a resurgence globally [25–27]. Antenatal syphilis screening and treatment coverage remains low in many countries [28, 29], resulting in numerous untreated or inadequately managed cases, [30–34] exacerbated by shortages of intramuscular benzathine penicillin-G (BPG), the only formulation currently recommended for treatment during pregnancy [35]. Gaps in the syphilis care cascade persist in both high- and low-income antenatal care (ANC) settings. Findings from a large cohort of pregnant WLHIV in Brazil demonstrated that 70% failed to receive the recommended syphilis screening during pregnancy. The study found women with detectable HIV RNA were more likely to receive syphilis screening suggesting a provider screening bias based on perceived risk of syphilis [36]. A study in the United States (U.S.) found 42% of pregnant individuals with syphilis between 2018 and 2021 were either inadequately treated or not treated at all. Limited access to timely prenatal care was identified as an important risk factor [37].

Staging syphilis to guide appropriate treatment during pregnancy is complex. WHO recommends a single dose of 2.4 million units (MU) BPG intramuscularly for early syphilis, and three weekly doses for late syphilis or syphilis of unknown duration [38]. Since many women are unsure when they acquired infection, the three-dose regimen is often recommended. Women must return weekly for treatment, and if a gap of >9 days occurs between doses it is recommended that the entire course be restarted [39], adding burden to women and health systems. Treatment should be initiated at least 30-days before delivery to reduce the risk of perinatal and neonatal complications [40], posing a challenge for women who initiate ANC late or become infected in their third trimester. Qualitative research among PLW, as well as healthcare providers revealed important barriers to effective antenatal syphilis screening, including inadequate provider training in syphilis management, lack of clear guidelines for antenatal syphilis screening, high testing costs, long distances to laboratories, late or no antenatal enrollment, and stigma [41, 42]. Untreated syphilis during pregnancy can lead to serious adverse outcomes including miscarriage, stillbirth, preterm birth, and congenital infection [43, 44]. Syphilis is a leading cause of preventable stillbirth globally [45].

Incidence of CS has been rising globally since the early 2000s [46, 47]. Between 2019 and 2023, incidence of maternal syphilis increased by 104% in the U.S., with a 106% increase in the incidence of CS. Between 2013 and 2023, the total number of reported CS cases increased by 740% (from 462 in 2013 to 3,882 cases in 2023), concurrent with a 580.8% increase (from 2.6/100,000 pregnant women in 2013 to 17.7/100,000 pregnant women in 2023) in primary and secondary syphilis among pregnant women. In 2023, 3,882 CS cases were reported in the U.S., including 279 CS-associated stillbirths, with an annual rate of 105.8 cases of CS/100,000 live births [48]. Similar trends have been observed in upper and LMICs [29]. In South Africa, maternal syphilis prevalence increased from 2.6% in 2019 to 3.2% in 2022 [49], with an increase in CS from 373 cases in 2020 to 1739 in 2023 [50, 51], in Malawi prevalence of maternal syphilis increased from 0.3% in 2014 to 1.9% in 2021 [52].

### Epidemiology of Syphilis and HIV co-infection in pregnancy

Between 2016 and 2021, the global pooled prevalence of HIV among pregnant women was 2.9%, with maternal syphilis estimated at 0.8%. In low-income countries (LICs), HIV prevalence was 5.2% and syphilis was 3.3%, while in LMICs, pooled prevalence of HIV and syphilis was 3.3% and 1.5% respectively [53]. Co-infection rates among pregnant WLHIV are limited, ranging from 0.05% to 10.2% globally [54]. Geographic heterogeneity in co-infection rates are driven by disparities in HIV and syphilis screening and diagnosis, socioeconomic conditions, individual risk factors, prevention programs, and access to ANC and treatment [54, 55]. Underreporting and inadequate surveillance, particularly in LMICs, lead to an underestimation of syphilis severity and hinder understanding of the burden and epidemiology of HIV/syphilis co-infection among people of reproductive age and in pregnancy [56, 57]. Early and sustained VS among pregnant women is strongly linked to a lower risk of HIV VT [58–61]. Genital ulcers, a symptom of primary syphilis, are linked to increased HIV transmission and acquisition [62]. Syphilis has been shown to double VT risk, with higher proportions of in-utero transmission among infants born to mothers with HIV/syphilis coinfection compared to those with HIV monoinfection [63].

Higher RPR titres at treatment and delivery are associated with an increased likelihood of syphilis VT [41]. Achieving serologic cure, a four-fold reduction in titre after adequate treatment, has been linked to younger age, higher baseline titres, and earlier stages of syphilis infection [64]. Associations between HIV status, CD4 count, and VL in WLHIV and syphilis serological outcomes remain inconsistent across studies [64] and limited among pregnant women. A 2022 study in Zambia observed that pregnant WLHIV showed greater reductions in RPR titres, faster rates of sero-reductions, and higher rates of serologic cure compared to pregnant women without HIV; [65] however, this study had a small sample size and could not control for syphilis treatment adherence between groups.

Lower socioeconomic status and educational levels, alcohol and drug use, and higher risk sexual behaviors increase the risk of HIV and syphilis among women of reproductive age [5, 55, 66]. While younger women are generally at higher risk for sexually transmitted infections (STIs), prevalence of HIV/syphilis co-infection during pregnancy is higher in women aged 25 years and older [67–69], potentially due to condomless sex and use of oral (and injectable) PrEP [54, 70]. Low rates of partner testing and treatment for both HIV and syphilis also contribute to the sustained risk of infection among pregnant women [71–74] and have been associated with worse birth outcomes [75].

## Strategies for improving HIV and Syphilis prevention and treatment in pregnancy

Tenets of VT elimination strategies for HIV and syphilis overlap but have varying success due to differences in the natural history of disease, transmission dynamics, diagnostic tests, and treatments (Table 1). These strategies include primary prevention of maternal infection, screening and diagnosis early and throughout ANC, curative treatment (syphilis) or VS for HIV soon after diagnosis, infant post-exposure prophylaxis, early infant diagnosis (EID) and treatment.

Point-of-care (POC) rapid diagnostic tests (RDTs) have revolutionized HIV and syphilis testing by providing quick results, typically 15–60 min [76, 77]. Cost-effectiveness [78] and diagnostic accuracy [79] of these tests have facilitated widespread use, decentralizing HIV and syphilis testing, improving case detection and timely treatment [80–82]. Timely ART and/or BPG dramatically reduces risk of VT and sequelae [83]. POC RDTs minimize loss to follow-up in LMICs, which has been associated with delays due to turnaround time with traditional laboratory methods [38]. Dual HIV/syphilis RDTs for initial screening in ANC for pregnant women without known HIV infection [84], can improve diagnosis, timely treatment and prevent adverse outcomes [85]. Uptake of routine HIV/syphilis dual testing in ANC has been slow, with laboratory-based syphilis testing standard of care in many countries [86]. Competing public health priorities and limited resources hinder government funding and decision-making for diagnostics. In 2021, cost of dual HIV/syphilis RDT was under US$1, addressing cost barriers and promoting broader adoption and implementation by governments [87]. Lack of well-developed guidelines, limited healthcare worker training, and availability of accurate and user-friendly tests remain barriers [88]. As of 2023, 62% of countries with data available in UNAIDS Global AIDS Monitoring (GAM) used laboratory-based testing as the routine method for syphilis screening in ANC [86].

Early detection and treatment of maternal HIV and syphilis are vital for reducing VT risk and associated complications [89, 90]. WHO recommends that all pregnant women undergo serological testing for both HIV and syphilis during their first antenatal visit, followed by immediate treatment. Repeat HIV and syphilis screening later in gestation is also recommended for women with an initial negative test [89, 91]. Partner treatment is essential for syphilis management, including expedited partner therapy (EPT).

WLHIV should begin lifelong ART immediately [92]. With early detection, ART, and comprehensive care for WLHIV, VT risk of HIV can be reduced to less than 1% [93–95]. Pregnant women with syphilis should receive immediate BPG [89]. When administered early in infection and pregnancy, BPG can prevent CS in 97% of cases, reduce stillbirths by 82%, preterm delivery by 64%, and neonatal death by 80% [96, 97].

## Progress toward strengthening HIV and Syphilis interventions for pregnant women and newborns

In 2014, WHO established global guidelines to eliminate VT of HIV and syphilis, expanding these in 2021 to include hepatitis B virus (HBV) under the"triple elimination initiative” [98]. This paradigm shift emphasizes moving away from siloed programming toward integration of prevention, detection and treatment within maternal-child health programmes. Considerable progress has been made toward elimination of HIV VT over the past 5 years, driven by primary and secondary prevention interventions (including PrEP), increased testing and ART coverage, and integration of HIV treatment into antenatal and postnatal care. While many VT programmes were designed around HIV interventions, notable progress has been made in integrating syphilis, with 79% of 149 countries with available data in UNAIDS GAM having national plans for eliminating VT of syphilis, and 87% of these plans integrating syphilis and HIV [86]. To date, 11 Latin American, 3 Asian, and 5 Eastern European countries have received WHO validation for elimination of VT of HIV and/or syphilis [99]. WHO’s Path to Elimination initiative provides a tier-based framework for achieving triple elimination in high burden countries [100], with Botswana and Namibia becoming the first high-burden countries certified on the path to HIV elimination. Gaps in the syphilis care continuum, limited availability of routine antenatal syphilis screening and treatment data, and challenges with CS case definitions and surveillance pose challenges to certification for syphilis. WHO developed a tool for national programmes to model CS cases, including stillbirths, and forecast testing and treatment needs [101]. The tool has been shown to improve country-level monitoring toward VT of syphilis [102], although estimates remain limited by gaps in national surveillance and diagnostic data used to parameterize the model.

## Advancement in research on HIV and Syphilis prevention, screening and treatment in pregnancy

### Screening and diagnostics

Syphilis diagnostic research is growing, which is critical for prompt and accurate detection and treatment during pregnancy. A recent systematic review found that treponemal POCT increased syphilis screening rates and reduced syphilis-related adverse pregnancy outcomes more effectively than no screening, laboratory-based RPR and TPHA screening, or non-treponemal POCTs [82]. Novel approaches to strengthening screening uptake among pregnant women are needed. A recent study of opt-out syphilis screening in the emergency department in Chicago increased pregnancy screening from 6 to 50%, and a 750% increase in diagnosed cases of active syphilis [103]. Because pregnant women in the U.S. often seek ANC in emergency departments [104], this highlights a differentiated approach to increasing syphilis screening at critical touchpoints. South Africa has moved to more frequent syphilis screening during pregnancy, aligned with the HIV testing schedule, including screening ≥ 4 times during pregnancy, with use of the dual HIV/syphilis test in women without HIV [105]. Use of the dual HIV/syphilis POCT facilitates testing for both infections with a single finger prick, but does require revision of existing HIV testing algorithms to ensure alignment of HIV/syphilis recommended screening frequencies. Test algorithms for pregnant WLHIV or who are newly diagnosed with HIV during pregnancy will also need to be tailored, particularly in a context like South Africa with high HIV prevalence and incidence among pregnant women. The revised screening strategy should be accompanied with healthcare provider training and supporting guidance, and evaluation of implementation outcomes.

Approaches to increasing access to timely ANC and strengthening testing implementation and uptake in these settings are urgently needed. In Kenya, HIV self-testing was feasible and acceptable to improve uptake of repeat HIV testing among PLW [106], highlighting the promise and importance of person-centred and female-controlled interventions. Early initiation of ANC was improved through a community-based home pregnancy screening that was acceptable among women, which enabled earlier screening and treatment [107]. A meta-analysis reported that detection of syphilis during the first or second trimester reduced the risk of preterm birth by 47%, stillbirth by 86%, and low-birth weight by 63% when compared to detection within the third trimester [108].

### Treatment and management

Community-based health care was effective in enhancing HIV-related health outcomes for mothers and children in low-resourced settings, particularly when integrated with facility-based approaches [109]. In Zambia, one-on-one counselling, home-based couples and male partner HIV testing, referrals to ART for male partners living with HIV, and appointment reminders via phone or SMS increased ART initiation and adherence among pregnant WLHIV [110]. Integrated, family-focused VT packages, including task-shifting, POC testing, integrated service provision for mother and infant, and community and male-partner engagement has improved maternal ART uptake and retention of mother-infant pairs [111]. WHO has updated its guideline to recommend POC VL testing for pregnant WLHIV [112]. POC VL testing has expanded testing access, minimized turnaround times, provided same-day results, and enabled timely clinical responses to elevated VL, thereby supporting continuous treatment and effective VS [113–115]. Innovative strategies to improve syphilis treatment completion and adherence among pregnant women are essential. Incorporating provider-focused behavioural interventions—engaging opinion leaders, client reminders, audits with feedback, supportive supervision, along with POCT kits—led to nearly 100% of pregnant women screened for syphilis and ensured treatment for all WLHIV. This approach achieved more than double the treatment rate compared to using POCT kits alone [116].

Novel approaches to strengthening partner engagement as a key strategy for prevention and management of maternal HIV and STIs are critically needed. In Kenya, Zambia, South Africa, and Uganda distribution of HIV self-testing (HIVST) for male partners of pregnant women was acceptable and effective at increasing partner testing rates when compared to clinic-based testing [117–120]. However, these studies also found gaps in linkage to ART among male partners, indicating intervention designs need to consider approaches for linkage between HIVST and ART initiation for male partners. In South Africa, HIVST together with adherence biofeedback counselling for pregnant women on PrEP and their partners was acceptable by pregnant women and led to substantial increases in both PrEP adherence among pregnant women and partner testing when compared to the standard of care [120], indicating the potential of HIVST for partners to also strengthen primary prevention of HIV in PLW. In Kenya, HIV testing and counselling advisors provided in-home testing for pregnant couples and educational support for serodiscordant couples, resulting in increased male partner testing and disclosure of HIV status and an increase in identification of serodiscordant couples [121]. EPT is widely recognized for its effectiveness in preventing recurrent STIs [122, 123]. A recent study that provided pregnant women who screened STI positive with options for partner treatment (contact slips, in-clinic treatment, or expedited partner therapy) demonstrated that EPT improved partner treatment [124]. Studies should explore novel approaches to administering injectable BPG in partner-friendly clinical or community spaces or interventions to improve adherence to the alternative regimen of 100 mg of doxycycline twice daily for 14-days.

### HIV early infant testing and diagnosis

Studies have demonstrated that POC HIV testing for EID is accurate, practical, and acceptable, and improves timely initiation of ART for HIV-positive infants when compared to standard-of-care centralised laboratory-based testing [125]. The effectiveness of differentiated models of service delivery for mother-infant pairs and EID has also been recently evaluated. Community-facility linkage models including Mentor Mother (mothers2 mothers [126]) and similar facility-based peer support models, decreased VT, improved EID rates, and reduced loss-to-follow-up, when compared to mother-infant pairs who received standard of care or no facility-community linkage support [127–130].

### Syphilis diagnostics in infants

Laboratory-based CS diagnosis is challenging because maternal treponemal antibodies cross the placenta during pregnancy and can persist for >5-months [131]. WHO CS case definition includes previously undiagnosed and untreated infants with non-reactive non-treponemal titres at ≥6-months [38]. As a result most guidelines suggest that syphilis-exposed infants have a non-treponemal test performed at birth as well as a careful examination looking for signs and symptoms of syphilis which present later. Clinical signs in the infant associated with in-utero HIV transmission may overlap with that of CS, for example infants may have microcephaly and hepatosplenomegaly at birth. A quantitative non-treponemal titre that is fourfold higher than the maternal titre is highly suggestive of CS, however a negative RPR does not exclude CS and most infected infants have titres that are the same or less than the mother’s titre. There are not commercially available treponemal IgM tests for use in infants, as sensitivity and specificity are suboptimal. Research into better diagnostics for CS is essential to adequately quantify burden and ensure optimal treatment. While WHO’s surveillance case definition for CS supports standardized reporting across countries, currently there is no agreed upon WHO definition for clinical diagnosis of CS.

#### Opportunities for integrated HIV and syphilis services in ante- and postnatal care

**Fig. 1:**
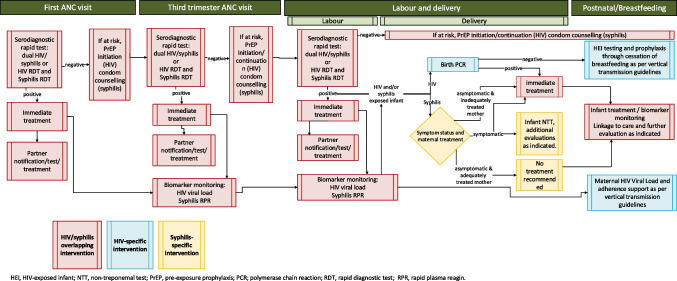
Overlap of HIV and syphilis services delivery and opportunities for improved integration for pregnant women and infants

Although specific services for prevention of HIV and syphilis differ, there are important interventions which overlap at key timepoints during ante and postnatal care (Fig. 1), presenting opportunities for bundling HIV and syphilis services across the care continuum **(**Table 1). Bundled HIV and syphilis counselling and testing, especially with POC diagnostics, allows simultaneous screening for both infections, increasing likelihood of early detection and timely treatment (same-day initiation or re-initiation of ART and BPG treatment for syphilis), which are critical in preventing VT of HIV and syphilis as well as adverse pregnancy and neonatal or infant morbidity and mortality [132]. Utilizing dual RDTs or multiplex tests for HIV and syphilis reduces the need for multiple visits and additional phlebotomy, lowering healthcare costs and minimizing burdens on both women and healthcare systems, especially in low-resource settings [85, 133].

**Table 1:** Opportunities for integrated HIV and syphilis prevention and treatment strategies for mother-infant pairs

	**Maternal**	**Infant**	**Maternal and infant**
Screening	**Decentralized Testing:** Implement point-of-care dual rapid diagnostic tests (RDTs) for HIV and syphilis at the first ANC visit to increase early detection of both infections. Incorporate screening and immediate treatment for other curable STIs **Repeat Testing:** Align HIV and syphilis retesting schedules, including in the third trimester and at labour/delivery, to identify and treat new infections	**Routine Newborn Screening**: Align screening/evaluation for infants at risk of CS to monitoring protocols for HIV-exposed infants including screening at birth, 6 weeks, 9 months (or immunization visit), and 18-month follow-up intervals	**Integration within broader contraception and PrEP services:** Integrating HIV and syphilis screening into contraception and PrEP services improves health outcomes for women and girls by enabling early detection and treatment (including screening of syphilis and HIV), aligning with PrEP services, preventing complications, and providing comprehensive sexual and reproductive health services **Differentiated Models for Service Delivery**: Increase access to home- or community-based screening for pregnant women and newborns in low-resource settings, such as through Community Health Workers, by incorporating combined screening and management of HIV and syphilis throughout pregnancy and the postnatal period
Diagnostics	**Multiplex Testing:** Scale-up the use of multiplex point-of-care diagnostics (e.g., dual HIV/syphilis RDTs in women without HIV) to screen for HIV and syphilis in the same sample, improving efficiency and cost-effectiveness	**Early Infant Diagnosis**: Align EID (including use of POC VL) with CS screening to improve immediate screening for HIV and syphilis in exposed infants	**Align maternal screening with infant screening** in maternal-infant pairs in service delivery (in immunization, antenatal care, and contraceptive services)
Management	**Integrated Management Protocols**: Integrate management protocols where WLHIV are routinely tested and immediately treated for syphilis given its frequent co-occurrence, ensuring co-management of both infections **Biomarker monitoring**: Package VL and treponemal titre value monitoring (where indicated), including at labour and delivery and 3–6 months after treatment and repeated if inadequate clinical or laboratory response to treatment **Partner Treatment**: Screen and treat partners of pregnant women for HIV, syphilis, and other curable STIs to prevent reinfection and improve outcomes for the mother and child, including expedited partner therapy **Linkage to Care:** Coordinate linkage-to-care strategies post-diagnosis, ensuring pregnant women with HIV and/or syphilis remain in care, adhere to treatment, and receive appropriate and comprehensive follow-up	**Integrated Management Protocols**: Where appropriate combine monitoring protocols for infants born to mothers with HIV and/or syphilis, ensuring they receive appropriate evaluation, treatment, and follow-up for both infections **Linking to Paediatric Care**: Coordinate referral pathways to ensure that infants exposed to HIV and/or syphilis are linked to comprehensive paediatric care, with long-term follow-up to monitor growth and development	**Postnatal Follow-Up Care:** Strengthen postnatal care for both the mother and the infant by integrating HIV and syphilis management into routine health check-ups, ensuring that both receive ongoing monitoring and treatment, as necessary **Linkage to Comprehensive Care:** For both mothers and infants, strong referral systems to specialized care (e.g., paediatric HIV or CS programs) ensure continuity of care and long-term health management for the family unit **Health Education**: Provide education for mothers and families about the importance of adherence to treatment regimens, repeat testing, and follow-up care for both mother and child **Community-Based Support**: Implement community-based interventions and differentiated models of service delivery which address both maternal and infant health, such as through Community Health Workers, Mentor Mothers, mobile clinics, or outreach programs, to ensure accessibility to prevention, treatment, and follow-up services
Surveillance systems	**Integrated ANC and Labour and Delivery Data Systems**: Integrate surveillance platforms that enable linkage of both HIV and syphilis infections (co-infection), treatment outcomes, VL, and treponemal titre values in pregnant women throughout ANC and labour/delivery	**Integrated Neonatal Surveillance**: Integrate surveillance systems that track both HIV and CS (and syphilis-associated birth complications) in newborns, enabling accurate data on co-infection cases, treatment outcomes, and follow-up care	**Case Management Systems:** Integrate case management systems which improve client-level service delivery and monitoring and provide healthcare workers with a comprehensive view of each woman’s medical history, HIV/syphilis/STI status, and social factors (e.g., partner support, socio-economic challenges, gender-based violence), allowing for person-centred care coordination and follow-up **Integrate mother-infant records**: Link ANC, labour and delivery, and postnatal records for mothers and their infants, including VL and/or treponemal titres, ensuring that infants born to mothers diagnosed with HIV and/or syphilis are screened and receive comprehensive follow-up to ensure early diagnosis and improve integration of care across both conditions **Outcome Monitoring**: Use surveillance data to monitor the success of interventions for both HIV and syphilis, such as ART, PrEP and syphilis treatment in preventing VT and adverse birth outcomes, and adapt strategies based on real-time monitoring and evaluation of outcome, need for improved definition of CS to adequately describe burden

Coordinated screening and management protocols, including at the first antenatal and third trimester visit, and labour/delivery enable coordinated testing, treatment, and follow-up, ensuring that pregnant WLHIV and/or syphilis receive comprehensive, prompt care throughout pregnancy and postpartum. Additionally, monitoring both VL and non-treponemal titres together, where indicated, supports personalized treatment adjustments and can support coordinated and streamlined health systems resources [134]. By consolidating records and case management for mother-infant pairs, healthcare providers gain a comprehensive view of each mother-infant pair, reducing fragmentation and ensuring that follow-up assessments and care are continuous and appropriate. Leveraging differentiated models of service delivery, including community and facility-community linkages, for HIV and syphilis services would facilitate access to person-centred care, making it easier for pregnant women, mother-infant pairs, and partners to receive essential health services within their communities. Integrating HIV and syphilis partner notification and treatment can help identify co-infection, reduce syphilis reinfection, and addresses broader family health needs. Counselling for HIV and syphilis should be provided to support disclosure, when ready, and support partner testing and treatment, including use of at-home HIV self-testing and referral for partner testing and treatment for syphilis and other STIs (e.g., EPT). There is a well-documented link between HIV/STI disclosure and an increased risk of intimate partner violence, [135–137] which can significantly hinder care-seeking and treatment adherence for pregnant women [138]. Partner notification and engagement strategies must be designed and implemented with the safety and wellbeing of women at the centre, including counselling on the risk that HIV or syphilis status disclosure may present. Findings from qualitative work in rural Kenya suggest that strategies should complement clinic services with community-based approaches such as encouraging male partners to take on a supportive role in maternal health and including male partners in health-related decisions such as antenatal HIV testing [137].

#### Challenges with integrating services

Effective service delivery within integrated HIV and syphilis programmes requires significant resources, including a well-trained health workforce, adequate diagnostic equipment and supplies, infrastructure readiness, and strong funding and policy support are needed. Workforce shortages (including unclear responsibilities for which providers deliver integrated HIV and syphilis care), and inconsistent access to diagnostic tests and treatments, particularly in rural areas, present major obstacles to equitable integration of HIV and syphilis services [41, 139]. Operational challenges—including insufficient provider training and refresher programs, unclear testing and screening guidelines, gaps in supportive supervision, frequent stockouts or expired test kits or BPG for syphilis, compounded with overburdened staff, and inadequate data tools to document test results and treatments—hinder the effective and sustainable implementation of integrated programs [140, 141]. There is concern about cost and cost-effectiveness of this resource-intensive comprehensive package of care. Implementation of additional services, including scale-up of screening tests, may incur downstream costs for end-users, in this case pregnant women. Providing free screening services in antenatal and postnatal care has been found to increase screening uptake [142]. Governments must address issues around governance of vertical programming at national and sub-national levels, resource allocation, and implications of bundled service provision on health systems and end-users to ensure equity, sustainability, and effectiveness of VT prevention [42–47, 49–151].

## Future Research Recommendations:

We recommend focused research, including implementation science studies, to improve the integration of syphilis and HIV in pregnancy, including:

1. Implementation science studies on how to improve integration of HIV and syphilis diagnostics and treatment.
2. Studies on improved diagnostics and treatment protocols for syphilis which could follow the model of HIV management, where a combination of early diagnosis and rapid ART initiation have contributed towards reduced VT.
3. Research on improved syphilis diagnostics for infants in the early neonatal period including IgM, PCR and novel antigen detection tests.
4. Safe, effective, and accessible treatment for pregnant women, minimizing VT risk and adverse pregnancy outcomes, including combination therapies e.g. long-acting penicillin and ART or PrEP combinations.
5. Integration of HIV and syphilis screening in contraceptive services including pre-conception services for women who wish to become pregnant for HIV testing, ART initiation, PrEP for prevention, syphilis screening and treatment.

## Supplementary Information

Below is the link to the electronic supplementary material.

Supplementary file1 (DOCX 30 KB)

## Data Availability

No datasets were generated or analysed during the current study.
