# Supplementary material for: Opportunities to Optimize Outcomes of Diagnosis and Treatment of HIV and Syphilis in Pregnancy: the Quest to Eliminate Maternal and Vertical Transmission
Source: Curr HIV/AIDS Rep. 2025 Apr 23;22(1):30. doi: 10.1007/s11904-025-00739-y (PMC12014709; doi:10.1007/s11904-025-00739-y)
Supplement: Supplementary file 1 — Supplementary file1 (DOCX 30 KB) [file 11904_2025_739_MOESM1_ESM.docx]

**Table 1. Opportunities for integrated HIV and syphilis prevention and treatment strategies for mother-infant pairs**

|  | **Maternal** | **Infant** | **Maternal and infant** |
| --- | --- | --- | --- |
| **Screening** | **Decentralized Testing:** Implement point-of-care dual rapid diagnostic tests (RDTs) for HIV and syphilis at the first ANC visit to increase early detection of both infections. Incorporate screening and immediate treatment for other curable STIs.  **Repeat Testing:** Align HIV and syphilis retesting schedules, including in the third trimester and at labour/delivery, to identify and treat new infections. | **Routine Newborn Screening**: Align screening/evaluation for infants at risk of CS to monitoring protocols for HIV-exposed infants including screening at birth, 6 weeks, 9 months (or immunization visit), and 18-month follow-up intervals. | **Integration within broader contraception and PrEP services:** Integrating HIV and syphilis screening into contraception and PrEP services improves health outcomes for women and girls by enabling early detection and treatment (including screening of syphilis and HIV), aligning with PrEP services, preventing complications, and providing comprehensive sexual and reproductive health services.  **Differentiated Models for Service Delivery**: Increase access to home- or community-based screening for pregnant women and newborns in low-resource settings, such as through Community Health Workers, by incorporating combined screening and management of HIV and syphilis throughout pregnancy and the postnatal period. |
| **Diagnostics** | **Multiplex Testing:** Scale-up the use of multiplex point-of-care diagnostics (e.g., dual HIV/syphilis RDTs in women without HIV) to screen for HIV and syphilis in the same sample, improving efficiency and cost-effectiveness. | **Early Infant Diagnosis**: Align EID (including use of POC VL) with CS screening to improve immediate screening for HIV and syphilis in exposed infants. | **Align maternal screening with infant screening** in maternal-infant pairs in service delivery (in immunization, antenatal care, and contraceptive services). |
| **Management** | **Integrated Management Protocols**: Integrate management protocols where WLHIV are routinely tested and immediately treated for syphilis given its frequent co-occurrence, ensuring co-management of both infections.  **Biomarker monitoring**: Package VL and treponemal titre value monitoring (where indicated), including at labour and delivery and 3-6 months after treatment and repeated if inadequate clinical or laboratory response to treatment.  **Partner Treatment**: Screen and treat partners of pregnant women for HIV, syphilis, and other curable STIs to prevent reinfection and improve outcomes for the mother and child, including expedited partner therapy.  **Linkage to Care:** Coordinate linkage-to-care strategies post-diagnosis, ensuring pregnant women with HIV and/or syphilis remain in care, adhere to treatment, and receive appropriate and comprehensive follow-up. | **Integrated Management Protocols**: Where appropriate combine monitoring protocols for infants born to mothers with HIV and/or syphilis, ensuring they receive appropriate evaluation, treatment, and follow-up for both infections.  **Linking to Paediatric Care**: Coordinate referral pathways to ensure that infants exposed to HIV and/or syphilis are linked to comprehensive paediatric care, with long-term follow-up to monitor growth and development. | **Postnatal Follow-Up Care:** Strengthen postnatal care for both the mother and the infant by integrating HIV and syphilis management into routine health check-ups, ensuring that both receive ongoing monitoring and treatment, as necessary.  **Linkage to Comprehensive Care:** For both mothers and infants, strong referral systems to specialized care (e.g., paediatric HIV or CS programs) ensure continuity of care and long-term health management for the family unit.  **Health Education**: Provide education for mothers and families about the importance of adherence to treatment regimens, repeat testing, and follow-up care for both mother and child.  **Community-Based Support**: Implement community-based interventions and differentiated models of service delivery which address both maternal and infant health, such as through Community Health Workers, Mentor Mothers, mobile clinics, or outreach programs, to ensure accessibility to prevention, treatment, and follow-up services. |
| **Surveillance systems** | **Integrated ANC and Labour and Delivery Data Systems**: Integrate surveillance platforms that enable linkage of both HIV and syphilis infections (co-infection), treatment outcomes, VL, and treponemal titre values in pregnant women throughout ANC and labour/delivery. | **Integrated Neonatal Surveillance**: Integrate surveillance systems that track both HIV and CS (and syphilis associated birth complications) in newborns, enabling accurate data on co-infection cases, treatment outcomes, and follow-up care. | **Case Management Systems:** Integrate case management systems which improve client-level service delivery and monitoring and provide healthcare workers with a comprehensive view of each woman’s medical history, HIV/syphilis/STI status, and social factors (e.g., partner support, socio-economic challenges, gender based violence), allowing for person-centred care coordination and follow-up.  **Integrate mother-infant records**: Link ANC, labour and delivery, and postnatal records for mothers and their infants, including VL and/or treponemal titres, ensuring that infants born to mothers diagnosed with HIV and/or syphilis are screened and receive comprehensive follow-up to ensure early diagnosis and improve integration of care across both conditions.  **Outcome Monitoring**: Use surveillance data to monitor the success of interventions for both HIV and syphilis, such as ART, PrEP and syphilis treatment in preventing VT and adverse birth outcomes, and adapt strategies based on real-time monitoring and evaluation of outcome, need for improved definition of CS to adequately describe burden |

**Supplemental Table 1. Current guidelines for diagnostics, treatment and management of HIV and syphilis in pregnancy**

| Stage of intervention | HIV | Syphilis |
| --- | --- | --- |
| Serodiagnostic testing during antenatal care | - HIV provider-initiated testing and counselling (PITC) at first ANC visit.^87^ - Retest all HIV-negative pregnant women in the third trimester, during labour, or postpartum In settings with a generalized HIV epidemic.^87^ - Repeat HIV testing in the third trimester for pregnant women who are at increased risk of acquiring HIV in settings without a generalized epidemic.^86^ - Additional retest at 14 weeks, 6 months or 9 months postpartum may be considered for women HIV.^137^ - Task-shifting using HIV RDTs for pregnant women.^87^ | - Screen all pregnant women at the first antenatal care visit.^80^ - Repeat screening test in the third trimester or labour/delivery for women with initial negative result and at elevated risk for infection.^138,139^ - Syphilis POC tests preferred in settings with high loss to follow-up or limited laboratory capacity.^36^ |
| Treatment or management of infected mother | - Lifelong ART for newly diagnosed women at any CD4 count. - Re-initiate ART for women who have been disengaged from care.^87^ - Dolutegravir (DTG)-based regimen combined with nucleoside reverse transcriptase inhibitors (NRTIs) like tenofovir and lamivudine or emtricitabine preferred. - Efavirenz (EFV)-based regimen acceptable alternative for pregnant women unable to use DTG.^126,140^ - VL test at first ANC visit for women already established on ART. - VL tested 3 months after initiation of ART for pregnant women newly diagnosed. - VL test at 34–36 weeks gestation, or delivery, for all pregnant WLHIV regardless of when ART was initiated. - VL tests 3 months after delivery and every 6 months thereafter for all breastfeeding WLHIV, regardless of when ART was initiated. ^106,137^ - Same-day POC VL testing to expedite result return and clinical decision-making.^137^ - CD4 counts should bechecked at first antenatal visit, with additional monitoring based on ART duration and immune status.^141^ - Screen for opportunistic infections, including TB, bacterial infections, STIs, and fungal infections including cryptococcosis (depending on local HIV prevalence and clinical indication)^142^ - Support for partner disclosure, including conseling and screening for risk of IPV.^127^ - Partner testing and treatment after disclousre.^127^ | - Immediate treatment with one dose of BPG 2.4 MU intramuscularly (IM) for early syphilis.^85^ - Late syphilis or infection of unknown duration, three doses of 2.4 MU BPG IM at one-week intervals one week apart.^36^ If more than 9 days pass between doses, the full treatment course must be restarted.^37^ - Pregnant women with a penicillin allergy should undergo desensitization before receiving BPG, as there are no effective alternatives to penicillin for treating syphilis in pregnancy.^36^ - Non-treponemal titers (NTT) repeated at 32 weeks and delivery (if at least 8 weeks after treatment) for syphilis diagnosed and treated at or before 24 weeks' gestation. If concern for reinfection or treatment failure ecists, earlier testing may be warranted - NTT titers checked again at delivery if syphilis is diagnosed and treated after 24 weeks gestation,.^37^ - For syphilis diagnosed in the second half of pregnancy, foetal ultrasound to assess for indicators of CS (e.g., hepatomegaly or foetal anaemia) without delaying treatment. If abnormalities are present, consultation with obstetric specialists is recommended, and a second dose of BPG may be beneficial for foetal health.^37^ - Support for partner disclosure, including conseling and screening for risk of IPV.^127^ - Partners testing and treatment regardless of symptom status.^85^ |
| Early infant testing and diagnosis | - POC nucleic acid testing (NAT) technologies (e.g, PCR) to diagnose HIV among infants and children younger than 18 months.^106^ - All HIV-exposed infants to receive virological testing as soon as possible including repeated NAT before 18 months and ELISA after the end of breastfeeding.^127,137^ - Positive NAT result should be followed by a confirmatory test to ensure accurate diagnosis^127,137^ |  |
| Infant treatment | - Breastfed HIV-exposed infants (HEI) of mothers on ART should receive six weeks of daily nevirapine (NVP) for prophylaxis.^137^   Four-to-six-week regimen of daily NVP or twice-daily zidovudine (AZT) is recommended ror HEI receiving replacement feeding.^137^  HEI at high risk of HIV transmission should receive a combination prophylaxis of twice-daily zidovudine (AZT) and daily nevirapine (NVP) for the first six weeks of life, regardless of feeding method (breastfed or formula-fed).^137^   - For high-risk breastfed HEI, HIV prophylaxis should be extended to 12 weeks, continuing with either a combination of twice-daily AZT and daily NVP or daily NVP alone.^137^ - Enhanced prophylaxis for all HEI may be beneficial in settings with low maternal ART coverage, high loss to follow-up rates, inadequate VL testing, and low maternal VS rates to prevent VT.^137^ - For infants with a positive HIV test, same-day linkage to care including ART with an age-appropriate ART regimen should be initiated immediately once the HIV diagnosis is made and while awaiting confirmation.^143^ | - No treatment is recommended for asymptomatic infants whose mothers had syphilis that was adequately treated with no signs of reinfection, however, infant with a reactive NTT should be followed serologically to ensure the result returns to negative^144,145^ - For infants who are asymptomatic and whose mothers had untreated or inadequately treated syphilis (including treatment within 30 days of delivery or with non-penicillin regimens), one dose of IM BPG 50 000U/kg is recommended.^144^ - For infants with confirmed or highly probable CS, treatment with 100,000–150,000 U/kg/day of aqueous benzyl penicillin IV or 50,000 U/kg/day of procaine penicillin IM, administered once daily for 10–15 days is recommended.^144,145^ - For CS, follow-up treatment of hearing and vision and neurodevelopment and check if titer falls. |
